# Histoplasmosis in Idaho and Montana, USA, 2012–2013

**DOI:** 10.3201/eid2106.141367

**Published:** 2015-06

**Authors:** Randall J. Nett, Donald Skillman, Laurel Riek, Brian Davis, Sky R. Blue, Elizabeth E. Sundberg, Joel R. Merriman, Christine G. Hahn, Benjamin J. Park

**Affiliations:** Montana Department of Public Health and Human Services, Helena, Montana, USA (R.J. Nett, J.R. Merriman);; Centers for Disease Control and Prevention, Atlanta, Georgia, USA (R.J. Nett, B.J. Park);; St. Peter’s Medical Group, Helena (D. Skillman);; Lewis and Clark City-County Health Department, Helena (L. Riek);; Billings Clinic, Billings, Montana (B. Davis);; Sawtooth Infectious Diseases, Boise, Idaho, USA (S.R. Blue);; Holy Rosary Healthcare, Miles City, Montana (E.E. Sundberg);; Idaho Department of Health and Welfare, Boise (C.G. Hahn)

**Keywords:** histoplasmosis, Idaho, Montana, endemic diseases, mycoses, fungi, United States, Histoplasma capsulatum

**To the Editor:** Histoplasmosis occurs after infection with the dimorphic fungus *Histoplasma capsulatum* (*1**–**6*). Patients become ill after they inhale soil contaminated with *H. capsulatum* (*1**,**2*). Most infections are asymptomatic or result in mild illness not determined to be histoplasmosis (*1**,**2*). Symptoms usually develop 3–14 days after exposure and range from self-limited pneumonia to severe disseminated disease requiring antifungal therapy (*2**,**7*).

In the United States, *H. capsulatum* is endemic to the Mississippi and Ohio River Valleys (*1**,**2**,**5**,**8*) but is not known to be endemic to the Rocky Mountain region (*8*). During June 2012–November 2013, a total of 6 unrelated cases of histoplasmosis were reported in Idaho (n = 1) and Montana (n = 5) in patients who had no recent travel to recognized *H. capsulatum*–endemic regions. Public health authorities investigated the illnesses by reviewing medical records and collecting exposure and travel histories.

The median age of the patients (3 male, 3 female) was 68 (range 17–79) years (Table). Each case was diagnosed by a different physician; no known epidemiologic links existed among the patients. Five patients had >1 immunocompromising conditions (Table), and 2 had acute pneumonia; 1 each had left parotid gland enlargement, anterior cervical lymphadenopathy, tricuspid valve mass, and acute changes in mental status. Three patients were hospitalized: 2 required intensive care, and 1 died.

**Table:** Characteristics of 6 persons with histoplasmosis, Idaho and Montana, USA, 2012–2013*

Characteristic	Value
Sex	
M	3 (50)
F	3 (50)
Median age, y (range)	68 (17–79)
Location of residence	
Idaho, southwestern	1 (17)
Montana	
Eastern	2 (33)
Southwestern	3 (50)
Immunocompromising condition, n = 5†	
Diabetes mellitus, type 2	3 (50)
Hepatitis C	1 (17)
Previous history of breast cancer	1 (17)
Acute mononucleosis	1 (17)
Previous history of colon cancer	1 (17)
Hospitalization	3 (50)
Death	1 (17)
Tests with positive results that contributed to histoplasmosis diagnosis	
Culture	2 (33)
Histopathology‡	2 (33)
Urine enzyme immunoassay‡	2 (33)
Diagnosis delayed >6 mo	3 (50)
At-risk activities	
Using potting soil containing bat guano	1 (17)
Exploring caves	1 (17)
Mowing grass in pasture	1 (17)
Cleaning pigeon cages	1 (17)
Traveling to an area where the disease is endemic <3 y of illness onset	0
None known	2 (33)

*Values are no. (%) patients except as indicated. †One patient had diabetes mellitus type 2 and hepatitis C; 1 patient had diabetes mellitus type 2 and a previous history of colon cancer. ‡One patient with a culture positive for *Histoplasma capsulatum* also had histopathology and urine enzyme immunoassay results consistent with *H. capsulatum* infection (results not shown).

Histoplasmosis was diagnosed primarily on the basis of culture (n = 2), urine enzyme immunoassay (EIA) (n = 2), and histopathologic examination (n = 2) results; histopathologic examinations were conducted by 2 pathologists (Technical Appendix Table). One patient with *H. capsulatum*–positive cultures also had positive results by histopathology, serum antigen detection, and urine EIA. Another patient with positive urine EIA results for antigen detection also had low serum levels of *Histoplasma* antibodies measured by compliment fixation. No patient samples were tested by PCR.

The interval between a patient’s initial visit to a health care provider and diagnosis ranged from 1 week to 20 months. Diagnosis was delayed >6 months for 3 patients. For 2 patients, a diagnosis was made on the basis of an *H. capsulatum*–positive urine EIA result <4 weeks from illness onset. Four (67%) patients underwent surgical procedures before histoplasmosis was diagnosed.

Each patient reported having traveled to *H. capsulatum*–endemic places, but none had traveled to these areas within 3 years of illness onset. Four patients reported exposures possibly related to infection (1 patient each): handling bat guano–containing potting soil manufactured in California, exploring caves, mowing pasture grass, and cleaning pigeon cages. The exposure to potting soil occurred in California; the other 3 exposures occurred in Montana. Two patients had no identifiable high-risk exposures to *H. capsulatum*.

These 6 patients with histoplasmosis represent potential acute infections and suggest that *H. capsulatum* might exist in Idaho and Montana, a geographic area farther west than areas where the fungus is known to be endemic. Areas of contaminated soils exist in microfoci outside recognized *H. capsulatum*–endemic areas and can be the source of infection for some persons (*6*). Previous studies suggest that the *H. capsulatum*–endemic area might extend into Montana and possibly other states in the Rocky Mountain region (*8**–**10*). Further environmental studies are needed to determine with certainty whether *H.* *capsulatum* fungi exist in natural environments in the Rocky Mountain region.

Delayed diagnosis of histoplasmosis increases the likelihood of delays in administering effective antifungal therapy. Histoplasmosis was diagnosed in 3 of these patients >6 months after they first sought care, probably because they had reported no recent travel to *H. capsulatum*–endemic areas. Among these 3 patients, none had urine EIA testing for the presence of *H. capsulatum* antigen. The 2 patients who received a diagnosis of histoplasmosis <4 weeks after they first sought care were assessed by using urine EIA. Urine EIA is a noninvasive and sensitive assay with high specificity but is subject to false-positive results in patients with other fungal infections, particularly blastomycosis (*6*), which is not known to be endemic in Montana or Idaho.

Investigation of these 6 histoplasmosis cases was limited because only 2 patients had cultures positive for *H. capsulatum,* and each patient had a remote travel history (>3 years before infection) to an *H. capsulatum*–endemic area. These limitations raise the possibility that the cases represent reactivation of latent disease or delayed clinical manifestations following a low-inoculum exposure years earlier in an area where the fungus is endemic (*2**,**6*). However, data supporting the possibility that reactivation of latent *H. capsulatum* infection causes acute illness are inconclusive (*6*).

In summary, health care providers should consider a diagnosis of histoplasmosis for Idaho and Montana residents having symptoms consistent with the disease, regardless of whether they have a travel history to recognized *H. capsulatum*–endemic areas. When considering a diagnosis of histoplasmosis, providers should also consider testing with urine EIA, a noninvasive way to assess the presence of *H. capsulatum* infection.

**Technical Appendix.** Laboratory findings in histoplasmosis patients, Idaho and Montana, USA, 2012–2013.
