# Supplementary material for: Histoplasmosis in Idaho and Montana, USA, 2012–2013
Source: Emerg Infect Dis. 2015 Jun;21(6):1071–2. doi: 10.3201/eid2106.141367 (PMC4451912; doi:10.3201/eid2106.141367)
Supplement: Supplementary file 1 — Technical Appendix. Laboratory findings in histoplasmosis patients, Idaho and Montana, USA, 2012–2013. [file 14-1367-Techapp-s1.pdf]

Article DOI: http://dx.doi.org/10.3201/eid2106.141367

Histoplasmosis in Idaho and Montana, USA, 2012–2013

Technical Appendix

Technical Appendix Table. Laboratory findings in histoplasmosis patients, Idaho and Montana, USA, 2012–2013*

Case no.	Residence	Year of symptom onset	Specimen source	Interval from symptom onset to testing	Culture results	Histopathology results	*H. capsulatum* antigen EIA	*H. capsulatum* antibody by compliment fixation
1	SW Idaho	2012	Urine	1			Positive	
			Blood	<1	No growth			
			Blood	2			Positive	
			Nasal tissue	2	*H. capsulatum*	Necrotizing granulomatous inflammation with rare budding yeast consistent with *H. capsulatum*		
2	SW Montana	2011	Urine	21			Negative	
			Parotid gland	20		Lymphoepithelial cyst and necrotizing granulomatous inflammation with rare fungal yeast forms consistent with *H. capsulatum*		
3	SW Montana	2011	Urine	14			Negative	
			Lymph node	13		Necrotizing granulomatous inflammation with yeast forms consistent with *H. capsulatum*		
4	SW Montana	2013	Urine	<1			Positive	
5	E Montana	2012	Lung tissue	6	*H. capsulatum*			
6	E Montana	2013	Urine	<1			Positive	
			Blood	<1				<1:8
			Blood	<1	No growth			

*E, eastern; EIA, enzyme immunoassay; *H. capsulatum, Histoplasma capsulatum;* SW, southwestern.
